# Supplementary material for: Decorin Promotes Osteoblastic Differentiation of Human Periodontal Ligament Stem Cells
Source: Molecules. 2022 Nov 25;27(23):8224. doi: 10.3390/molecules27238224 (PMC9739490; doi:10.3390/molecules27238224)
Supplement: Supplementary file 1 [file molecules-27-08224-s001.zip › molecules-1970961-supplementary.pdf]

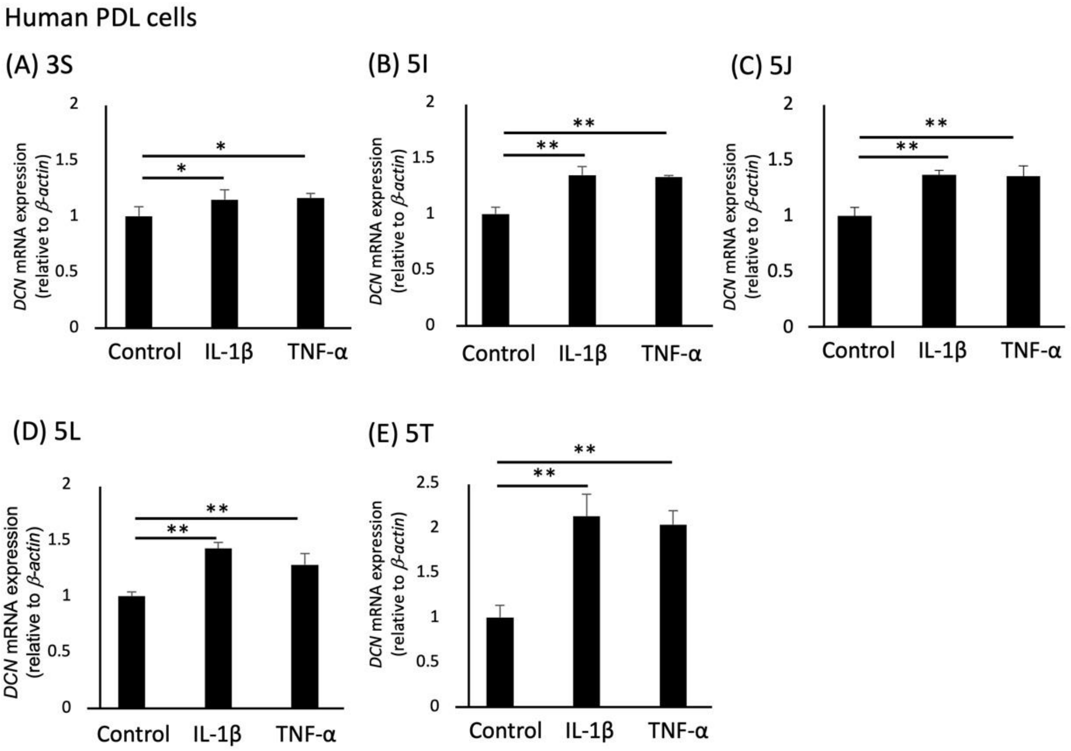

Supplemental Figure S1. Effects of IL-1β and TNF-*α* on *DCN* expression in human PDL cells.

Gene expression of*DCN* in human PDL cells 3S(A), 5I (B), 5J (C), 5L (D) and 5T (E) treated with IL-1β (10 ng/ml) and TNF-α (10 ng/ml) for 6 h (A–E) was examined by quantitative RT-PCR. Untreated cells were used as the control. Normalization was performed against *β-actin* expression. Gene expression levels are shown as the fold increase relative to the control. Values are the mean ± SD of three independent experiments.

**$p < 0.01$.

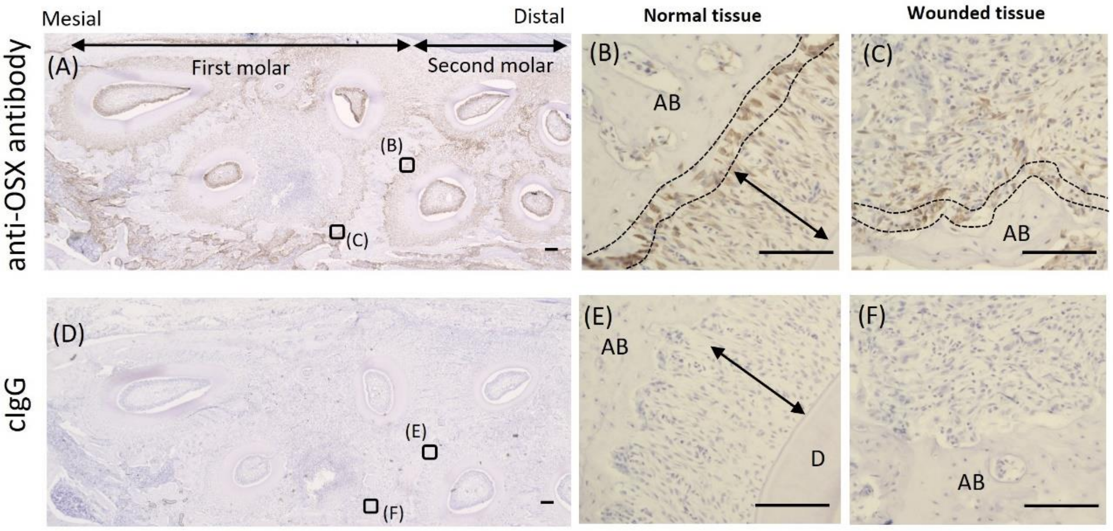

Supplemental Figure S2. Expression of OSX in wounded rat periodontal tissue.

(A-F) Immunochemical analysis of OSX expression was performed in wounded rat periodontal tissue, including PDL tissue and alveolar bone. (A-C)Localization of anti-OSX antibody-positive areas in the normal site (B) and wounded site (C) were examined on days 5 after surgery. Panels B, C, E, and F were magnified images of panels A and D. Control IgG was used for the negative control (D-F). Nuclei were counterstained with hematoxylin. Double arrow indicates PDL tissue. Inside of two dotted lines exhibited anti-OSX-positive areas. D, dentin; AB, alveolar bone. Bars = 100 μm.

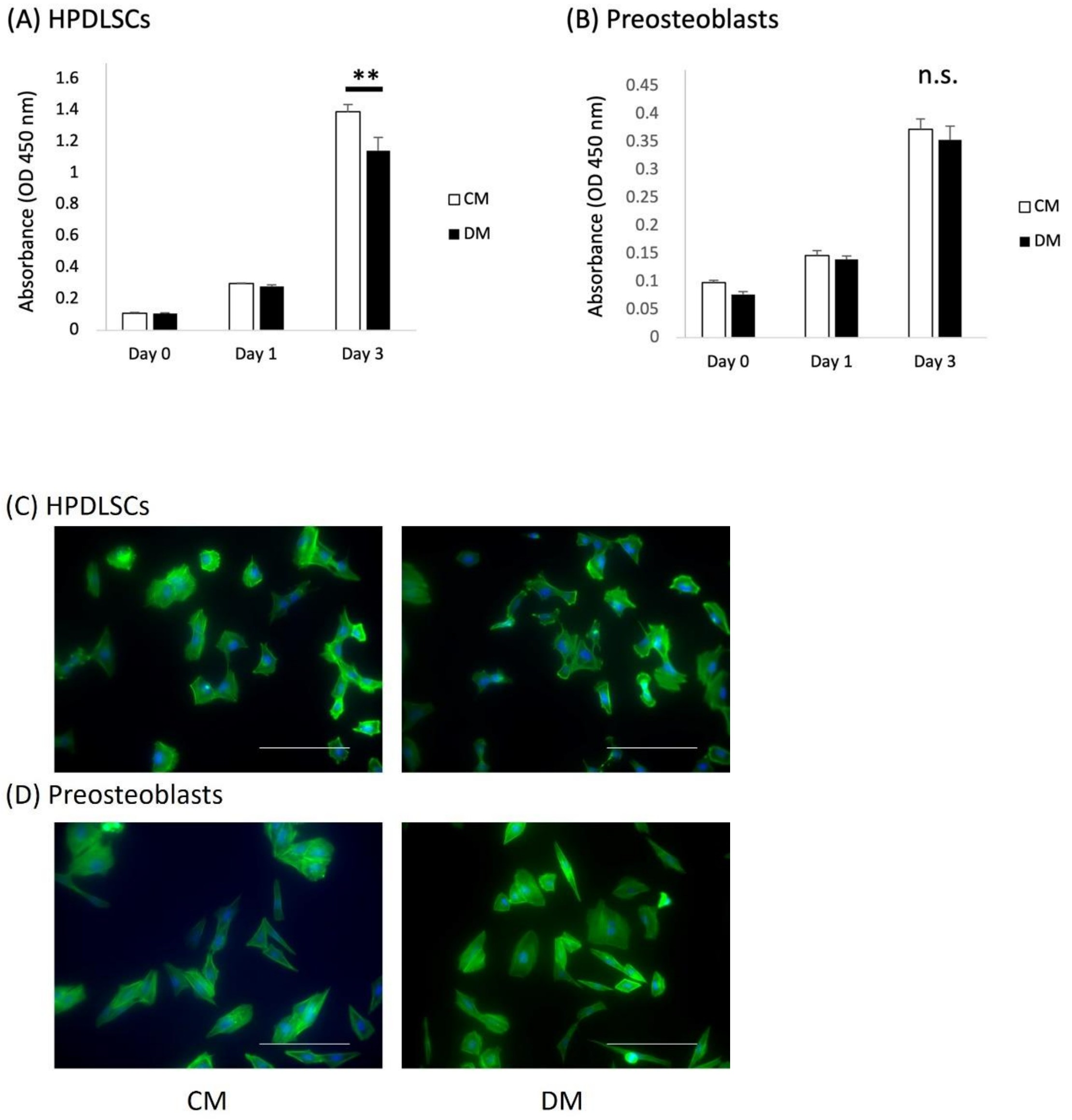

Supplemental Figure S3. Effects of $CaCl_2$ treatment on proliferation and morphology of HPDLSCs and preosteoblasts

(A, B) HPDLSCs (A) and preosteoblasts (B) were cultured in 10% FBS/α-MEM (CM) or CM with 1.5 mM $CaCl_2$ (DM) for 0, 1, and 3 days. A proliferation assay was performed using the WST-1 proliferation assay kit at an absorbance of 450 nm. Values are the mean ± SD of three independent experiments. $**p < 0.01$, n.s. = no significance. (C, D) HPDLSCs (C) and preosteoblasts (D) were cultured in CM or DM for 24 hours for the cytoskeletal staining. Bars = 200μm.
